# Atypical chronic myeloid leukemia found in a patient with eosinophilia for six years: a case report

**DOI:** 10.1186/s12877-024-05196-7

**Published:** 2024-07-11

**Authors:** Moqin Jiang, Meng Chen, Lixiang Yan, Ying Zhang, Xiangdong Yang, Weifeng Zhang

**Affiliations:** 1 Department of Hematology, First Teaching Hospital of Tianjin University of Traditional Chinese Medicine, Tianjin, 300381 China; 2 National Clinical Research Center for Chinese Medicine Acupuncture and Moxibustion, Tianjin, 300381 China

**Keywords:** Atypical chronic myeloid leukemia, MDS/MPN with neutrophilia, Eosinophilia, Molecular genetics, Myelodysplastic/myeloproliferative neoplasms

## Abstract

**Background:**

Atypical chronic myeloid leukemia (aCML) is a highly aggressive type of blood cancer that falls under the category of myelodysplastic/myeloproliferative neoplasms (MDS/MPN). In the fifth edition of the WHO classification of tumors, this category has been renamed MDS/MPN with neutrophilia. Although eosinophilia is commonly observed in blood cancers, it is rarely seen in aCML.

**Case presentation:**

This study presents a case of aCML that was diagnosed six years after the patient developed eosinophilia. The patient had undergone tests to rule out other primary and secondary diseases, but the eosinophilia remained unexplained. Treatment with corticosteroids and hydroxyurea had proven ineffective. Six years later, the patient experienced an increase in white blood cells, primarily neutrophils. After ruling out other possible diagnoses, a combination of morphologic and molecular genetic findings led to the diagnosis of aCML. The patient responded well to treatment with azacitidine.

**Conclusions:**

This study summarizes the current state of aCML diagnosis and management and discusses the possible connection between eosinophilia and aCML.

## Background

Atypical chronic myeloid leukemia (aCML) is a rare and aggressive hematopoietic stem cell malignancy. Its incidence is approximately 1–2 cases per 100 cases of *BCR::ABL1*−positive chronic myeloid leukemia (CML) [1, 2]. Clinically and biologically heterogeneous, aCML carries a high risk of progression to acute myeloid leukemia (AML) and a poor prognosis due to the lack of standardized treatment protocols. Due to overlapping bone marrow (BM) developmental abnormalities and hyperplastic features, aCML is classified as a subtype of myelodysplastic/myeloproliferative neoplasms (MDS/MPN) [3]. The term “aCML” was retained in the 2022 International Consensus Classification (ICC) of Myeloid Neoplasms and Acute Leukemia and was redefined in the 5th edition of the World Health Organization (WHO) classification of tumors of hematopoietic and lymphoid tissues as MDS/MPN with neutrophilia [3, 4].

The characteristics of aCML are indicated by white blood cell (WBC) counts ≥13 × 10^9^/L with increased neutrophils (NEU) and dysplasia, as well as immature BM cells in ≥10% of the WBC, with eosinophils (EOS) and monocytes usually being present in less than 10%. In recent years, molecular genetics has become a diagnostic focus in recent years to exclude other causes of clonal and reactive NEU. Mutations in *SETBP1*, *ASXL1*, and *ETNK1* are generally considered relevant for diagnosing aCML. In contrast, *BCR::ABL1* or tyrosine kinase fusions associated with myeloid/lymphoid neoplasms with eosinophilia, as well as *JAK2*, *MPL*, and *CALR* mutations, are used as exclusion criteria. The WHO diagnostic criteria also exclude cases with *CSF3R* mutations and those categorized as MDS/MPN with ring sideroblasts and thrombocytosis (MDS/MPN−RS−T) with *SF3B1* mutations.

Eosinophilia is commonly observed in hematologic neoplasms, but its association with aCML has been relatively underexplored. In this study, we present a case of a patient diagnosed with aCML harboring *ASXL1*, *SETBP1*, and *NRAS* mutations, six years after the onset of eosinophilia. The patient responded favorably to treatment with azacitidine (AZA), resulting in a significant reduction in end−organ damage from eosinophil infiltration.

## Case presentation

**Fig. 1:**
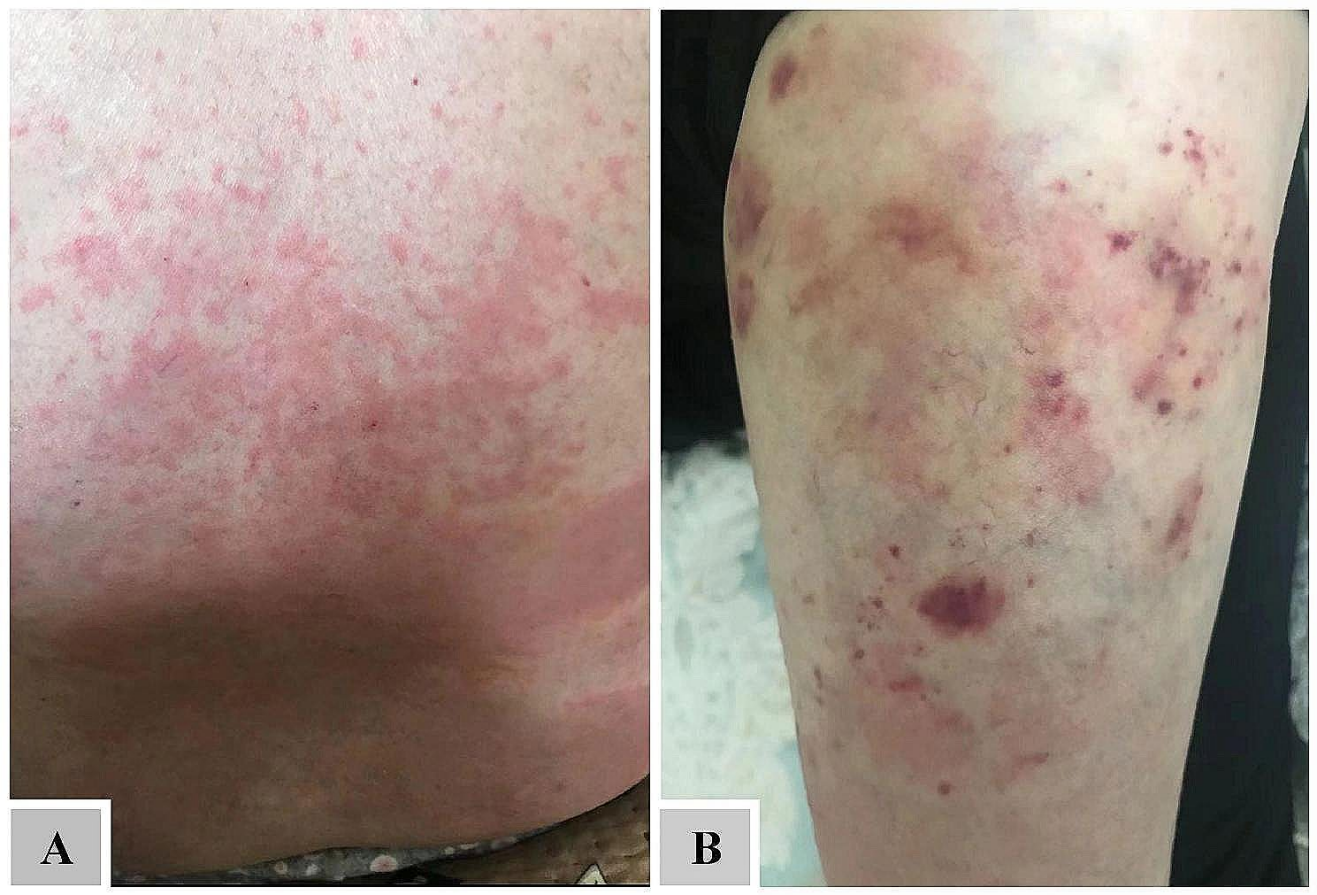
**(A)** The patient has had a recurrent rash and eosinophilia for six years. **(B)** The patient exhibited petechiae and ecchymoses on their lower extremities during their readmission for cerebral infarction in February 2021

A 68−year−old female patient who had multiple acute cerebral infarctions in 2016 presented to the hospital with an elevated EOS count of 1.97 × 10^9^/L. No abnormalities were found in NEU, WBC, red blood cell (RBC), hemoglobin (HGB), platelets (PLT), monocytes (MON), or basophils (BOS), and she received only symptomatic treatment for the multiple cerebral infarctions. Over the subsequent six years, the patient consistently had an elevated EOS count, but recurrent skin rashes with pruritus were not taken seriously (Fig. 1) until February 2021, when she was admitted to the hospital for another acute cerebral infarction with symptoms of chest tightness, breath−holding, flushing of the cheeks, and scattered bleeding spots on the skin. Peripheral blood examination showed an elevated WBC count of 11.44 × 10^9^/L, a RBC count of 6.93 × 10^12^/L, a HGB level of 167 g/L, a PLT count of 97 × 10^9^/L, a NEU count of 8.71 × 10^9^/L, a NEU percentage of 76.2%, an EOS count of 0.53 × 10^9^/L, an EOS percentage of 4.6%, a BOS count of 0.45 × 10^9^/L, a BOS percentage of 4%, a MON count of 1.59 × 10^9^/L, and a MON percentage of 13.9%. Immune system examination revealed that the patient had elevated complement C3 and C4, weakly positive anti−Jo−1 antibodies, and negative screens for rheumatoid factor, antinuclear antibodies, anti−double−stranded DNA antibodies, anti−histone antibodies, lupus anticoagulant, and anticardiolipin antibodies. Tests for non−hepatotropic viruses, such as fine virus antibodies, cytomegalovirus antibodies, and anti−EBV antibodies, were normal. Cranial and cardiac MR showed signs of eosinophilia with polyneurological involvement and EOS endocarditis.

**Fig. 2:**
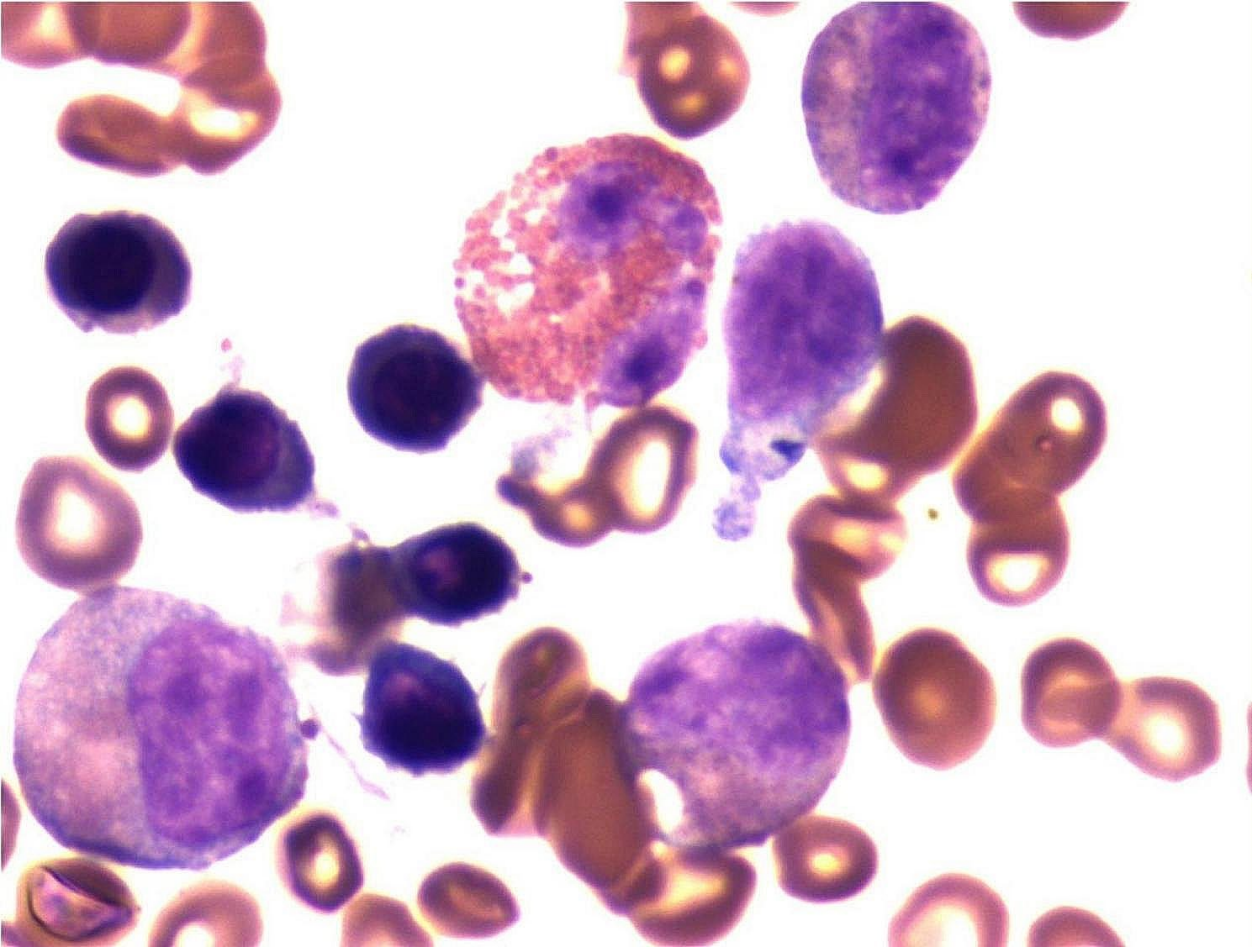
Dysplastic granulocytes and eosinophils were observed in the images of the patient’s bone marrow aspirate smear

Physical examination and abdominal ultrasound showed an enlarged spleen and a PET−CT scan revealed diffusely increased BM metabolism, indicating a possible hematologic lesion. Suspecting a myeloid tumor associated with eosinophilia, a BM evaluation was subsequently performed. BM Morphologic examination revealed granulocyte and megakaryocyte hyperplasia with trilineage developmental abnormalities (Fig. 2), including 1% primitive granulocytes, 0.5% early granulocytes, 13.5% intermediate granulocytes, 10.5% late granulocytes, 7% EOS, 7.5% lymphocytes, 26.5% nucleated RBCs, and 3% MON. The histologic evaluation of bone marrow reveals a satisfactory degree of myeloproliferation, marked by relatively high expression of Lysozyme, while the expression of E−Cadherin and CD235a is relatively low. Various stages of granulocytic and erythroid lineages are evident without significant deviation in their proportions. Megakaryocytes display positive staining for CD61, and both their quantity and morphology appear unremarkable. Flow cytometric immunofluorescence analysis revealed approximately 12.00% EOS and 0.84% early−stage myeloid cells, partially expressing CD33, CD117, CD64, CD11b, and CD13, but not expressing CD10, CD7, CD34, CD19, CD14, CD71, CD235a, CD56, or CD16. Abnormal expression of CD13/CD11b and CD13/CD16 differentiation antigens was observed, and no evidence of abnormal immunophenotype associated with acute leukemia, high−risk MDS, lymphoma, or myeloma was detected. The karyotype was normal. To rule out clonal eosinophilia and other myeloproliferative neoplasms, 56 fusion genes (including *BCR::ABL1*, *FIP1L1*, *PDGFRA*, *PDGFRB*, *JAK2::V617F*, etc.) were screened using RT−PCR, and all results were negative. The patient was initiated on anticoagulation therapy with a daily regimen of methylprednisolone 40 mg and continuous oral hydroxyurea.

**Fig. 3:**
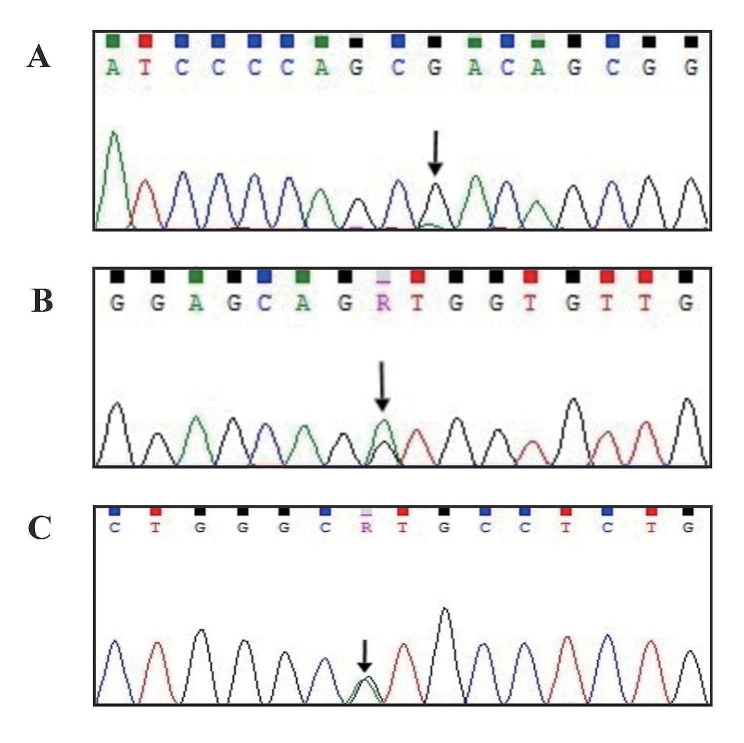
**(A)** PCR and gene sequencing of the *SETBP1* gene in this patient identified the variant on exon 4 (c.2602G > A). **(B)** PCR and gene sequencing of the *NRAS* gene in this patient identified the variant on exon 2 (c.35G > A). **(C)** PCR and gene sequencing of the *CSF3R* gene of this patient revealed the variant on the intron surrounding exon 5 (c.485 + 71 A > G)

Despite receiving treatment with corticosteroids and hydroxyurea, the patient’s condition deteriorated, and their WBC count, NEU count, and EOS count gradually increased. Consequently, methylprednisolone and hydroxyurea were gradually reduced and gradually discontinued. In July 2022, the patient presented with worsening central nervous system deficits, delirium, chest tightness, shortness of breath, and flushed cheeks and was subsequently admitted to the hematology department of our hospital. Upon admission, the patient’s WBC count was 61.21 × 10^9^/L, RBC count was 6.57 × 10^12^/L, HGB was 130 g/L, PLT count was 406 × 10^9^/L, NEU count was 50.51 × 10^9^/L, with a NEU percentage of 82.5%, EOS count was 5.41 × 10^9^/L, with an EOS percentage of 8.8%, BOS count was 0.29 × 10^9^/L, with a BOS percentage of 0.5%, MON count was 1.18 × 10^9^/L, and the percentage of MON was 1.9%. Peripheral blood smear analysis indicated an increased NEU percentage of 88%. Additionally, serum biochemistry showed an elevated lactate dehydrogenase (LDH) level of 425.8 U/L. Genetic analysis of the peripheral blood identified a heterozygous nonsense mutation at c.2338 C> T (p.Gln780Ter) in the *ASXL1* gene, a missense mutation at c.2602G> A (p.D868N) in the *SETBP1* gene, and a missense mutation at c.35G> A (p.G12D) in the *NRAS* gene. Furthermore, single nucleotide polymorphic site mutations were detected in the *CSF3R* gene, which was not considered pathogenic (Fig. 3). Based on the 2022 WHO diagnostic criteria, the patient was diagnosed with aCML with *ASXL1*, *SETBP1*, and *NRAS* mutations.

On July 18, 2022, the patient received hypomethylating therapy combined with a cytoreductive regimen, specifically AZA 100 mg d1–5 and cyclophosphamide 600 mg d2 and d5. The patient subsequently received a second course of chemotherapy with AZA 100 mg d1–5 on September 17, 2022, and a third course of AZA 100 mg d1–7 chemotherapy on October 27, 2022. Although the patient refused to repeat the BM exam, her WBC and NEU counts improved, while her EOS and PLT counts remained stable. Clinically, the patient’s splenomegaly resolved, her rash subsided, and her delirium improved. However, she did not return for further treatment over the subsequent four months and ultimately succumbed to multiple organ failure in February 2023.

## Discussion

Chronic and persistent eosinophilia can affect all organ systems and may even lead to life−threatening end−organ damage [5]. Therefore, identifying the underlying cause of eosinophilia is of immense clinical significance, and appropriate therapy should be initiated promptly to minimize further end−organ damage. Clonal eosinophilia is often associated with chronic myeloid neoplasms, such as MPN or MDS/MPN [6]. In the present case, the patient’s six−year history of eosinophilia complicated the clinician’s assessment, making it difficult to differentiate between symptoms of eosinophilic infiltration and immune system disorders affecting multiple organs. After ruling out the hypothesis of non−hematologic origin, testing for gene rearrangements associated with clonal eosinophilia, such as *BCR::ABL1*, *PDGFRA*, *PDGFRB*, *FGFR1*, *PCM1::JAK2*, and *JAK2::V617F*, did not yield any definitive clues. Conventional treatments for eosinophilia, including corticosteroids and hydroxyurea, did not significantly benefit the patient [7]. It was not until a genomic study was performed that the patient was found to harbor concurrent *ASXL1*, *SETBP1*, and *NRAS* mutations, which, combined with other morphological and laboratory features, supported a definitive diagnosis of aCML.

The diagnosis of aCML should exclude other myeloid malignancies with definite genetic lesions. Its differential diagnosis includes *BCR::ABL1* negative CML and other MPNs such as chronic neutrophilic leukemia (CNL) and chronic eosinophilic leukemia. The overlapping proliferative and developmental abnormalities observed in aCML pose challenges in distinguishing it from other MDS/MPNs such as chronic myelomonocytic leukemia and MDS/MPN, not otherwise specified (MDS/MPN−NOS), a potential challenge. Among these, differentiating aCML from MDS/MPN−NOS presents the most formidable challenge, particularly in the absence of molecular features within the diagnostic criteria [7, 8].

In the past, the diagnosis of aCML was mainly based on clinical morphology. However, in recent years, new insights into the molecular genetics of MDS/MPNs have significantly augmented our comprehension of aCML and shifted the focus toward the role of molecular genetics in its diagnosis [9]. Virtually all aCML patients harbor genetic mutations affecting growth factor signaling, transcriptional regulation, RNA splicing, and DNA methylation/histone modification pathways. However, owing to the inherent heterogeneity of aCML and the absence of distinct biomarkers, there is no single genetic alteration that is specific to the disease.

As sequencing technologies rapidly develop in scope and sensitivity, methodologies for detecting genetic alterations. Data derived from Next−Generation Sequencing (NGS) platforms has emerged as a pivotal tool in the differential diagnosis of myeloid neoplasms, augmenting the traditional assessment based on morphological and laboratory criteria.

Analysis of commonly mutated genes in aCML can determine determining clonality and facilitating the differentiation between tumorigenic and non−tumorigenic increases in WBC count [10]. The study found that *ASXL1* mutations occur at a high frequency (60–90%) in aCML, with genes such as *SETBP1*, *ETNK1*, *TET2*, *SRSF2*, *NRAS*, and *U2AF1* also being frequently mutated in aCML [1, 11]. Among these complex mutation patterns, simultaneous *ASXL1* and *SETBP1* mutations were most frequently observed, followed by simultaneous *SRSF2* and *SETBP1* mutations [11, 12]. Furthermore, investigations into clonal gene architecture indicate that *ASXL1* mutations typically manifest as early events in aCML, whereas mutations in *RAS*, *CBL*, *TET2*, *SRSF2*, and *SETBP1* tend to occur later in disease progression [1, 12].

Current research provides limited understanding about the clonal advantage of identified mutations in aCML, and further investigation is required to elucidate the clonal mechanisms of *ASXL1*, *SETBP1*, *NRAS*, and *CSF3R* gene mutations in relation to aCML progression and transformation. Among these mutations, *ASXL1* mutations have been found to trigger neutrophil dysplasia and have the potential to progress to myeloid malignancy [12]. *SETBP1* mutations are present in approximately one-quarter of aCML cases and play a role in apoptosis, transcription, and nucleosome assembly [13]. Compared to patients with wild−type *SETBP1*, those harboring *SETBP1* mutations have significantly elevated WBC counts, more severe thrombocytopenia and anemia, as well as more pronounced cellular dysplasia [14]. Mutations in genes related to the RAS/MAPK pathway are considered poor prognostic factors in MDS, CMML, primary BM fibrosis, and AML and be associated with leukemic transformation in aCML [11]. *CSF3R* mutations are considered drivers of leukemia and have been found in patients with aCML [15]. In a small sample study, Maxson et al. [15] found that more than half of patients with CNL or aCML harbored *CSF3R* mutations. Given the rarity of *CSF3R* mutations in other hematologic cancers, they are considered diagnostic features in patients with CNL and aCML. The 5th edition of the WHO classification emphasizes that the presence of a *CSF3R* mutation at the time of diagnosis of aCML should be critically reviewed to exclude an alternative diagnosis of CNL [3].

There is a lack of prospective data on the treatment of patients with aCML, and uniform treatment guidelines are unavailable. However, proliferative features, including increased WBC counts, splenomegaly, and associated somatic symptoms, can be effectively controlled with hydroxyurea over a short period. The management of anemia and red cell transfusion dependence is similar to that of MDS, utilizing erythropoietic agents and red cell transfusions. Hematopoietic stem cell transplantation (HSCT) is currently the only treatment associated with an improved prognosis, but it is associated with high relapse and mortality rates and is not preferred for the vast majority of patients [1].

Hypomethylating agents (HMAs) such as AZA and decitabine are widely used in aCML. Some data suggest that HMAs have the highest response rate in treating patients with aCML, with an overall response rate (ORR) of up to 33.3%. However, the duration of response is limited to an average of 1.7 months [8, 10]. Consequently, HMAs cannot be considered the standard of care for aCML. Instead, they are typically used as a bridge treatment for patients eligible for HSCT or as a stand−alone treatment for those who cannot undergo HSCT or for whom no clinical trial options are available [16].

Many unmet needs exist in patients with aCML. Current treatment strategies do not significantly improve the overall survival (OS) of aCML patients, with a median survival of only 20 months [10]. Precision genomic analysis helps assess the genetic and functional characteristics of aCML and identify personalized targeted therapeutic regimens [17]. Given the genetic polymorphism of *CSF3R* and its involvement in the JAK−STAT signaling pathway, JAK inhibitors (e.g., fedratinib and ruxolitinib) have garnered significant attention for the treatment of aCML. As a JAK inhibitor, fedratinib has been approved for use in patients with high−risk myelofibrosis. Recently, a multicenter, phase 2 study of fedratinib included 10 patients, one of whom had aCML. The results demonstrated favorable clinical efficacy of fedratinib in patients with proliferative features of MDS/MPN and CNL. Compared to ruxolitinib, fedratinib exhibits broader kinase inhibition profile, potentially enhancing its efficacy in high−risk, molecularly complex diseases [18].

Moreover, MEK inhibitors (e.g., trametinib) and SRC kinase inhibitors (e.g., dasatinib) have been evaluated in prospective clinical trials for patients with aCML [19]. Additionally, numerous ongoing trials are actively investigating the effects of combination therapies (e.g., AZA combined with venetoclax) for the treatment of aCML.

There is no consensus risk−prognosis stratification for aCML. Patnaik et al. [20] developed a risk−prognosis model for aCML and found that advanced age, low HGB, and *TET2* mutations were independent poor prognostic factors for aCML. Another study showed that age, PLT count, BM cell ratio, and LDH level were independent predictors of survival [11]. In molecular genetics, a synergistic effect of various molecular events contributes to poor outcomes in aCML. Among them, *ASXL1* mutations in aCML were not significantly associated with the prognosis. *SRSF2* was associated with a better prognosis, while *RUNX1*, *NRAS*, and *CUX1* mutations were associated with a shorter OS [12]. Two retrospective studiesinvestigated the role of *SETBP1* mutations in the prognosis of aCML but reached different conclusions, possibly limited by the small number of cases [9, 12].

To date, the association between eosinophilia and aCML remains unclear. Determining the precise mechanism underlying the development of early eosinophilia in these patients is challenging, and it is difficult to ascertain whether eosinophilia triggers the development of aCML or occurs independently. Some extremely rare fusion genes, such as *CSNK2A1::PDGFRB*, *CBFB::MYH11*, and *NSD3::NUTM1*, have been identified in patients with eosinophilia using RNA−seq techniques. We speculate that the patient in this case may have certain extremely rare molecular genetic changes that closely link eosinophilia to aCML. Therefore, we recommend performing genomic studies as early as possible in patients with eosinophilia of unknown cause to clarify the type and period of disease onset.

It is worth noting that the EOS counts in our patient were consistently higher than usual. However, in a small percentage of patients, EOS counts may be elevated even when the EOS percentage is within the normal range, especially when the WBC is high. Currently, the ICC diagnostic criteria suggest that the percentage of EOS in aCML should be less than 10%, while the WHO diagnostic criteria do not provide a clear requirement for EOS. Patients with aCML and increased EOS counts may represent a novel diagnostic category within the spectrum of MDS/MPN disease spectrum.

## Conclusions

Our findings provide preliminary evidence of a specific association between aCML, a rare disease, and eosinophilia. Following the exclusion of other underlying conditions, aCML should be considered as a potential diagnosis in patients presenting with eosinophilia. Hence, there is an urgent imperative for studies to probe the correlation between eosinophilia and aCML. An in−depth exploration of the underlying mechanisms may help clinicians control the progression of the disease in the early stages of eosinophilia and potentially stop the onset of an aggressive disease like aCML.

## Data Availability

The datasets used and/or analysed during the current study are available from the corresponding author on reasonable request.

## Abbreviations

aCML: Atypical chronic myeloid leukemia
MDS/MPN: Myelodysplastic/myeloproliferative neoplasms
CML: Chronic myeloid leukemia
AML: Acute myeloid leukemia
WHO: World Health Organization
WBC: White blood cell
NEU: Neutrophils
EOS: Eosinophils
AZA: Azacitidine
RBC: Red blood cell
HGB: Hemoglobin
PLT: Platelets
MON: Monocytes
BOS: Basophils
LDH: Lactate dehydrogenase
CNL: Chronic neutrophilic leukemia
HSCT: Hematopoietic stem cell transplantation
HMAs: Hypomethylating agents
OS: Overall survival
